# Electrical characterization and examination of temperature-induced degradation of metastable Ge_0.81_Sn_0.19_ nanowires†
†Electronic supplementary information (ESI) available: A potential failure mechanism and estimation of diffusion lengths is described. In addition, XRD patterns, EDX maps as well as line scans. See DOI: 10.1039/c8nr05296d


**DOI:** 10.1039/c8nr05296d

**Published:** 2018-10-12

**Authors:** M. Sistani, M. S. Seifner, M. G. Bartmann, J. Smoliner, A. Lugstein, S. Barth

**Affiliations:** a TU Wien , Institute of Solid State Electronics , Floragasse 7 , 1040 Vienna , Austria; b TU Wien , Institute of Materials Chemistry , Getreidemarkt 9 , 1060 Vienna , Austria . Email: sven.barth@tuwien.ac.at

## Abstract

Metastable germanium–tin alloys are promising materials for optoelectronics and optics. Here we present the first electrical characterization of highly crystalline Ge_0.81_Sn_0.19_ nanowires grown in a solution-based process. The investigated Ge_0.81_Sn_0.19_ nanowires reveal ohmic behavior with resistivity of the nanowire material in the range of ∼1 × 10^–4^ Ω m. The temperature-dependent resistivity measurements demonstrate the semiconducting behavior. Moreover, failure of devices upon heating to moderate temperatures initiating material degradation has been investigated to illustrate that characterization and device operation of these highly metastable materials have to be carefully conducted.

## Abstract 2

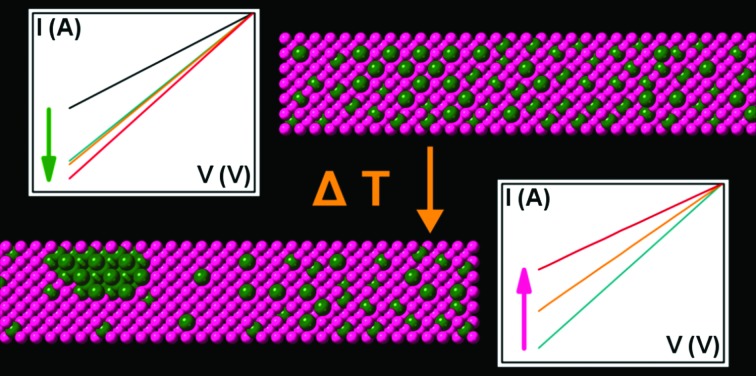
Electrical characterization of Ge_0.81_Sn_0.19_ nanowires has been performed revealing high electrical conductivity and semiconductor behaviour when cooled to 10 K. The impact on slightly elevated temperatures on the device stability of this metastable material is described.

## Introduction

Group IV semiconductor nanowires (NWs) are promising building blocks for various fields of application including electronic and sensing devices,^1^,^2^ solar cells,^3^,^4^ lithium ion batteries,^5^,^6^ *etc*. Their electronic properties can be altered by incorporation of well-known dopants in the semiconductor host lattice,^7^–^9^ while recently extraordinary high amounts of these known dopants as well as non-common metal incorporation in nanoscaled group IV elements is described.^10^–^13^ To date, the performance of Si- and Ge-based materials in optics and photonics is limited by the dominating, intrinsic indirect bandgap of their thermodynamically most stable allotropes with diamond cubic crystal structures.

A direct bandgap material based on Ge can be obtained by physical strain engineering in the semiconductor^14^–^16^ or an effective alloying with high Sn concentrations of ∼8–10 at% in Ge_1–*x*_Sn_*x*_,^17^,^18^ which exceeds the thermodynamic solubility limit (∼1 at% Sn).^19^ Since these Ge_1–*x*_Sn_*x*_ alloys are isostructural with Si and thus compatible with CMOS processing, this material is a very promising candidate for optoelectronics and optical devices operating in the infrared spectral region, such as lasers,^17^,^20^–^22^ photodetectors,^23^,^24^ light emitting diodes^25^–^27^ or biological sensors.^28^ Moreover, the electronic properties are also altered upon Sn incorporation in the Ge host lattice which should result in an enhanced electron and hole mobility making Ge_1–*x*_Sn_*x*_ interesting for high-speed electronics.^29^–^33^ Besides a large body of data related to the thin film growth on single crystalline substrates and significant recent advancements in this field,^17^,^22^,^34^–^36^ the number of reports on one-dimensional nanostructures and nanoparticles with significant Sn incorporation is still very limited.^37^ Top-down approaches based on the post-growth etching of epitaxial films to prepare desired morphologies have been applied^38^ and a few reports describing suitable bottom-up approaches for a reliable formation of Ge_1–*x*_Sn_*x*_ nanoparticles^39^–^43^ and one-dimensional nanostructures^44^–^50^ are available. Morphological control to obtain shape anisotropic single crystalline materials has been achieved using Ge NWs as templates in order to obtain core–shell Ge/Ge_1–*x*_Sn_*x*_ NWs^45^ but also non-templated metal-seed supported growth of Ge_1–*x*_Sn_*x*_ NWs *via* gas-phase^46^,^47^ and solution-based synthesis^44^,^48^,^49^ has been described in literature. To date, anisotropic Ge_1–*x*_Sn_*x*_ nanostructures usually are in the range of 9–13 at% Sn,^45^,^46^ while our microwave-based synthesis procedures gives access to highly crystalline material with very high Sn content of 17–32 at%.^44^,^49^ In general, literature data related to electronic properties of Ge_1–*x*_Sn_*x*_ materials with higher tin contents (>5 at%) are scarce and the provided mobility, charge carrier density or sheet resistance cannot be used to calculate the respective resistivity values, because either the required data are missing, thin films are strained or the material is p- or n-doped.^51^,^52^ This paper describes for the first time the electronic properties of bottom-up grown Ge_1–*x*_Sn_*x*_ NWs integrated in two-point and four-point configuration, revealing very high conductivity values while still retaining semiconducting properties. The Ge_0.81_Sn_0.19_ NWs electronic properties have been investigated in the temperature range of 10–298 K. In addition, the behavior of the devices when exposed to elevated temperatures is investigated emulating potential heating effects during device operation.

## Experimental

All synthetic procedures and handling of the chemicals for the nanostructure synthesis have been carried out using Schlenk techniques or an argon-filled glove box (MBraun). Butyl lithium, hexamethyldisilazane, SnCl_2_, 1,1,3,3-tetramethyldisilioxane, and GeCl_4_ were purchased from Sigma-Aldrich. All solvents for the precursor synthesis were dried using standard procedures and stored over molecular sieve. Precursors and intermediates have been prepared as described before.^49^ Dodecylamine (98%, Sigma-Aldrich) was distilled three times under reduced pressure using additions of 0.5–1 mL of Sn(N(Si(CH_3_)_3_)_2_)_2_ as described in literature.^44^ This procedure allows separation of impurities that can react with Sn(N(Si(CH_3_)_3_)_2_)_2_ and Ge(N(Si(CH_3_)_3_)_2_)_2_ in the following material synthesis and ensures the most reliable results.

### Nanostructure synthesis and temperature treatment

Ge_0.81_Sn_0.19_ NWs were synthesized in 10 mL glass cells (Anton Paar GmbH) at 503 K. In a typical experiment, 3 mL of dodecylamine were transferred in a glass microwave reactor. First, Sn(N(Si(CH_3_)_3_)_2_)_2_ and subsequently Ge(N(Si(CH_3_)_3_)_2_)_2_ were added to dodecylamine in a Sn : Ge ratio of 1 : 4. The mixture was then heated to ∼373 K and stirred at room temperature for 15–17 h. Further information about the pretreatment procedure of the precursor mixture for the synthesis of Ge_1–*x*_Sn_*x*_ NWs has been described in literature.^44^,^49^ The vial was sealed with a Teflon-coated cap and transferred to the microwave reactor (Monowave 300; Anton Paar GmbH; frequency, 2.46 GHz) equipped with an IR temperature control unit. The vessel was heated up as quick as possible, held 2–10 min at 503 K and finally was cooled down by a gas stream. The synthesized Ge_0.81_Sn_0.19_ NWs were collected by adding toluene (3 mL) and subsequent centrifugation. The NW material was redispersed in solvent (2× toluene; 3× ethanol, 3× toluene), centrifuged, separated from the supernatant and finally stored under ambient conditions in toluene.

Heat treatment of the NW samples has been carried out using a home-build CVD oven operated at 523 K under helium atmosphere. Before the samples have been heated up, the chamber has been purged by evacuation to 0.1 mbar and replacing the atmosphere by He 5.0. During the annealing a constant flow of 50 sccm He was channeled through the oven.

### Nanostructure characterization

Scanning electron microscope images have been acquired using a FEI Inspect F50. Ge_0.81_Sn_0.19_ NWs were deposited on lacey carbon copper grids (Plano) by drop casting of a toluene suspension for transmission electron microscope (TEM) characterization. In this study, a FEI TECNAI F20 operated at 200 kV and equipped with high angle annular dark field (HAADF) STEM and EDX detector was used. The EDX elemental maps and point measurements were recorded and quantified using the AMETEK TEAM package. The TEM images were recorded and treated using Digital Micrograph software.

X-ray diffraction (XRD) patterns were recorded on a PANalytical X-Pert PRO PW 3050/60 in Bragg–Brentano geometry using Cu-Kα radiation, while the analysis of the acquired data was performed using X-pert Highscore software. The preparation included drop casting of the nanowire material onto Si (911) wafers as support.

### Electrical characterization

The Ge_0.81_Sn_0.19_ NWs have been deposited onto a highly p-doped Si substrate with a 100 nm thick, thermally grown SiO_2_ layer and predefined macroscopic Ti–Au bonding pads. The devices have been prepared by electron beam lithography on a Raith e-LiNE machine (10 kV, PMMA resist) and individual NWs have been contacted with 7 nm Ti and 190 nm thick Au pads by electron beam evaporation using a Leybold e-beam evaporator. The metal pad evaporation was preceded by a short oxygen plasma treatment (300 W, 90 s; Technics plasma GmbH 100-e plasma system) to remove any organic shell and a subsequent HI dip was used to remove germanium oxide. The excess metal has been removed by standard lift-off techniques. Essentially, contacts to the NWs were prepared using established NW processing techniques.^53^,^54^

The electrical measurements at room-temperature and ambient conditions were performed using a combination of a semiconductor analyzer (HP 4156B) and a probe station. To minimize the influence of ambient light as well as electromagnetic fields, the probe station was placed in a dark box. The resolution limit of the used setup is 500 fA and leakage currents of ∼1 pA, which is negligible for the here investigated highly conducting material. Low-temperature measurements (10–298 K) were performed in vacuum at a background pressure of approximately 2.5 × 10^–5^ mbar using a ^4^He cryostat (Cryo Industries CRC-102) and a semiconductor analyzer (Keysight B1500A).

## Results and discussion

**Fig. 1:**
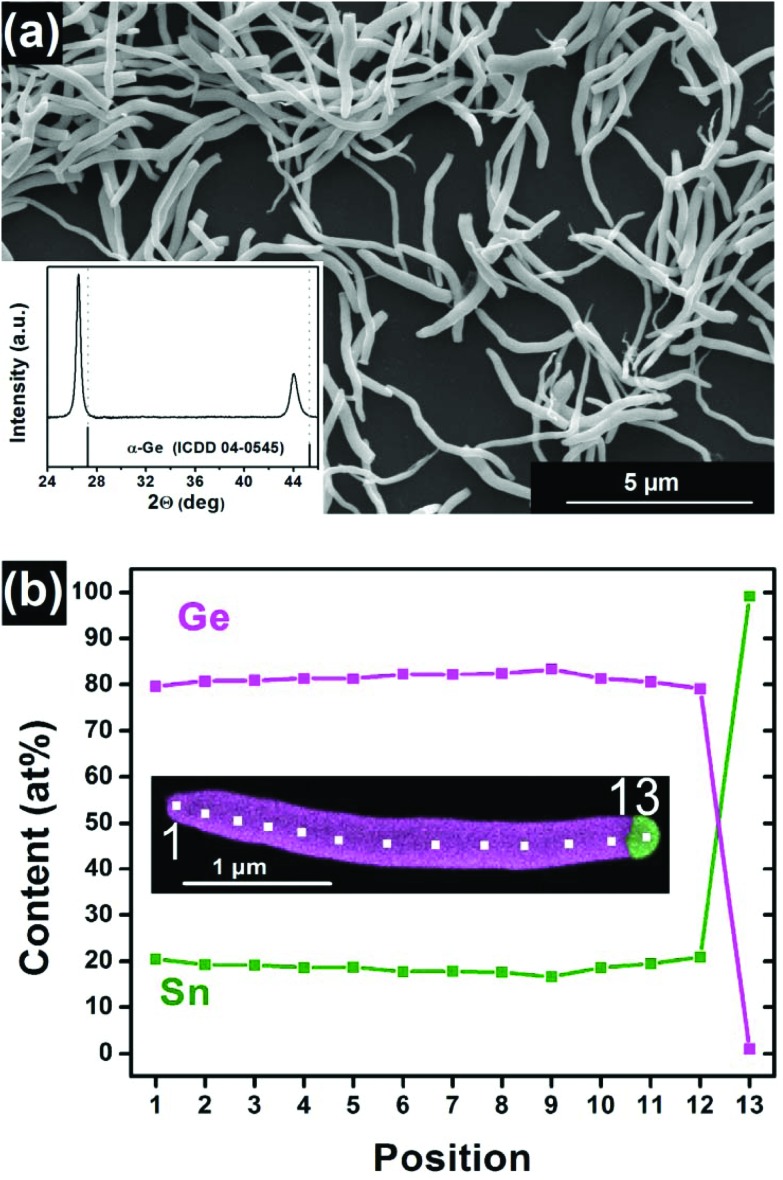
(a) SEM image of Ge_1–*x*_Sn_*x*_ NWs and corresponding XRD pattern (inset) after Sn seed removal, showing the shifted signal when compared to the Ge reference. (b) The composition of the Ge_1–*x*_Sn_*x*_ NW can be also determined by EDX point measurements while the specific locations along the NW are illustrated in the STEM-EDX image. The EDX map also shows the Sn growth seed used to form these nanostructures.

The Ge_1–*x*_Sn_*x*_ NWs have been prepared by a microwave-based synthesis procedure described in literature.^44^,^49^ A description of the process is provided in the Experimental section. Fig. 1a shows a scanning electron micrograph (SEM) image of the prepared NWs after treatment with hydrochloric acid to remove metallic growth seeds resulting in a pure, unaltered Ge_1–*x*_Sn_*x*_ material. The composition has been calculated from the X-ray diffraction (XRD) pattern (inset in Fig. 1a) according to Vegard's law using the isostructural α-Ge and α-Sn references. The obtained shift of the Ge_1–*x*_Sn_*x*_ reflections can be associated to 18.8 at% Sn. Scanning transmission electron microscopy energy dispersive X-ray spectroscopy (STEM-EDX) maps and line scans are shown in Fig. 1b illustrating a homogeneous distribution of Sn in the Ge matrix with only small fluctuations. Fig. 1b also indicates the metallic Sn growth promoter, which has been removed from all other NWs by HCl treatment for this study. Evaluation of EDX measurement data reveals 18.8 ± 1.2 at% Sn in the Ge_1–*x*_Sn_*x*_ NWs. Both values from EDX and XRD analysis are in good agreement and consequently, the material will be referred to as Ge_0.81_Sn_0.19_ NWs.

**Fig. 2:**
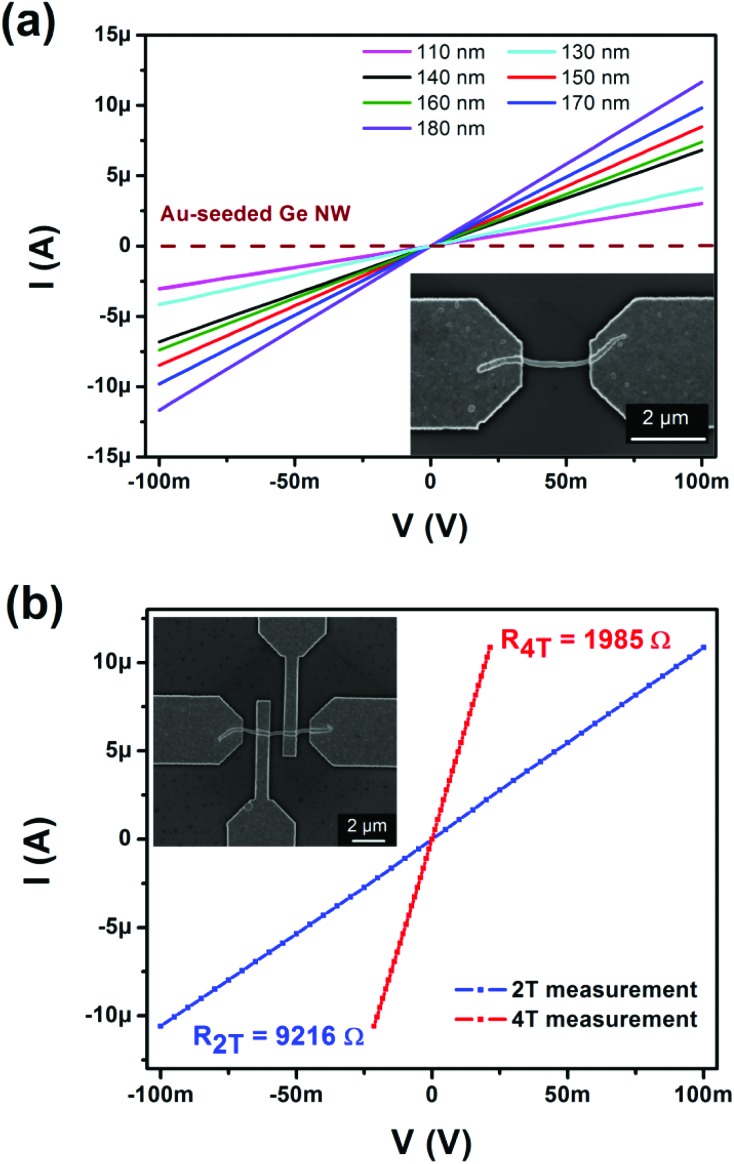
(a) Two-terminal *I*–*V* measurements of Ge_0.81_Sn_0.19_ NWs with diameters between 110–180 nm and a Au-seeded NW of intrinsic Ge (dashed line) for comparison. (b) Four-terminal devices are used to illustrate the influence of the contact resistance and the corresponding two-terminal measurement using the same NW is illustrated for comparison.

The electronic properties of the NWs have been investigated in two different geometries on Si substrates with a 100 nm thick, thermally grown SiO_2_ layer. The drop casted Ge_0.81_Sn_0.19_ NWs are contacted by gold pads using standard electron-beam lithography, deposition of metal by evaporation, and lift-off techniques. A specifically small or optimized contact resistance either through formation of interfacial layers based on Ni typically requires thermal annealing (≥623 K)^55^ or based on Sn electrodes with low Schottky barrier height^56^ have been neglected, because the required annealing temperatures or destabilization of the Ge_1–*x*_Sn_*x*_ material by the metallic contact can lead to undesired material degradation.^44^ However, even the Ge_0.81_Sn_0.19_ NW-based two-terminal devices show ohmic behavior combined with high current levels as can be expected for a semiconductor material with high number and mobility of charge carriers (Fig. 2a). A fluctuation of the resistance values of different devices while diameters in the range of 110–180 nm has been observed with thicker diameters showing typically higher conduction values. The electrical current is two orders of magnitude higher in comparison to intrinsic Ge NW with similar dimension grown by Au mediated CVD as shown in Fig. 2a. In contrast to slightly strained Ge_0.86_Sn_0.14_ as a high Sn content material,^57^ the here presented highly conductive Ge_0.81_Sn_0.19_ NW devices do not show any gating effect in field effect measurements (between –40 V and 40 V).

In order to investigate the influence of the contacts on the actual NW resistance values, four-terminal devices using individual NWs have been prepared. The inset of Fig. 2b shows such a device using a Ge_0.81_Sn_0.19_ NW of 170 nm thickness. The resistance measured in two-point geometry is about 9.2 kΩ, while the actual resistance of the NW determined in four-probe configuration is only 1.985 kΩ. In general, the devices showed a decrease in resistance to 22–24% of the values determined in two-point configuration. This clearly indicates a high contact resistance between the NW material and the Au contacts, that might be caused by the pretreatment of the NWs with an oxygen plasma to remove any organic groups from the surface, but at the same time oxidizing some of the Ge_0.81_Sn_0.19_ material at the surface and causing SnO_2_ formation that cannot be removed easily in the further process steps.

**Fig. 3:**
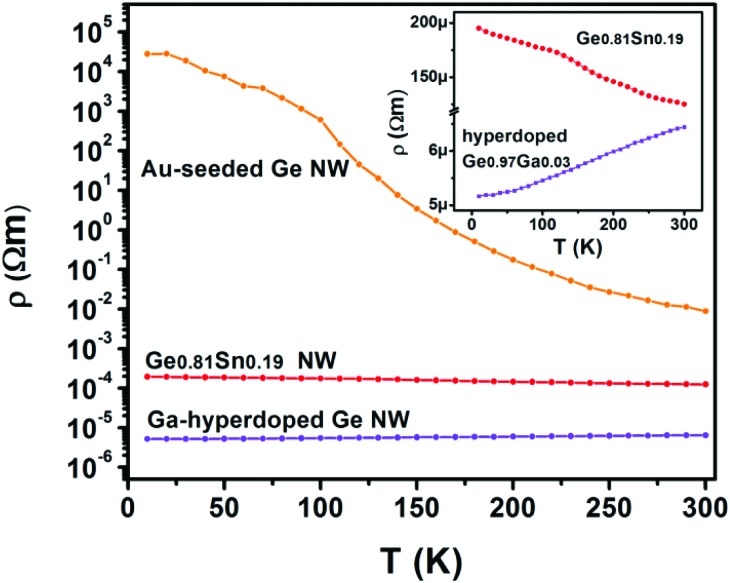
Evaluation of resistivity changes with temperature in the range 298–10 K for a Ge_0.81_Sn_0.19_ NW and comparison to other NWs including intrinsic Au-seeded Ge and Ga-hyperdoped Ge.

Fig. 3 illustrates the resistivity-dependence on temperature variation in the range from 298 to 10 K, which was investigated in four-point configuration. At room temperature, resistivity values of Ge_0.81_Sn_0.19_ NWs (∼1 × 10^–4^ Ω m) are typically 2 orders of magnitude lower than for intrinsic Ge (∼9 × 10^–3^ Ω m),^58^ but approximately two orders of magnitude higher than for hyperdoped Ge_0.97_Ga_0.03_ NWs (∼3 × 10^–6^ Ω m)^11^ as shown in Fig. 3. Theoretical predictions consider a significant increase in electron mobility for Ge_1–*x*_Sn_*x*_ with high tin content when compared to Ge (∼50 times for *x* = 0.2),^59^ while the charge carrier concentration increases only slightly according to studies on thin films of Ge and Ge_1–*x*_Sn_*x*_ with up to 5.8 at% Sn.^60^ This corresponds considerably well with the observed difference in resistivity values between intrinsic Ge and our Ge_0.81_Sn_0.19_ NWs assuming other parameters such as surface scattering would be similar on both types of NWs. Intrinsic Ge NWs grown by Au-seeding show a strong dependence on the temperature and an increase of resistivity by several orders of magnitude upon cooling. In contrast, the Ge_0.81_Sn_0.19_ NW's resistivity appear to be almost independent on temperature, similar to hyperdoped Ge_0.97_Ga_0.03_ NWs^11^ as described in literature. A more detailed view reveals an increase in resistivity with decreasing temperatures (inset Fig. 3) typical for a semiconductor while in comparison hyperdoped Ge_0.97_Ga_0.03_ NWs show quasi-metallic behavior with decreasing resistivity. This change in resistivity and the curve shape is expected for a semiconductor; however, the resistivity increases merely by ∼50–60% of the room temperature value when cooled to 10 K.

**Fig. 4:**
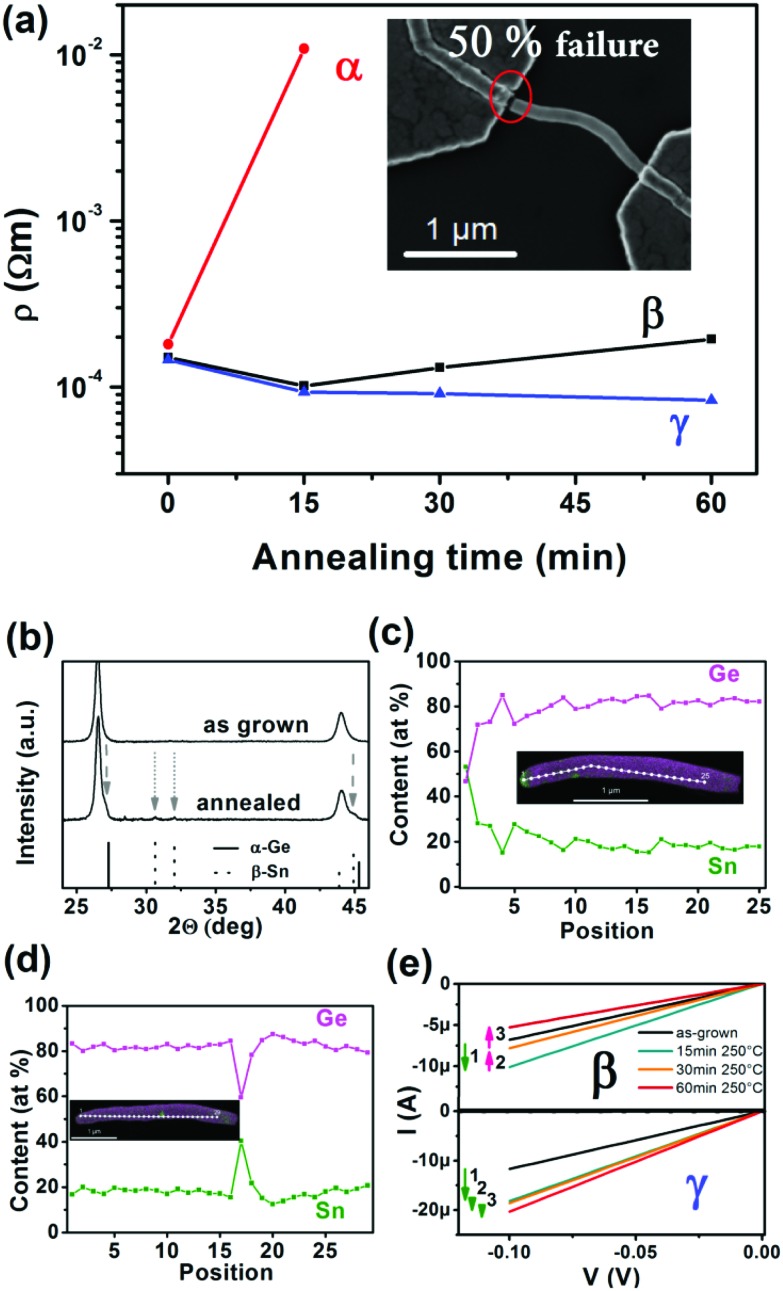
(a) Electronic behavior of two-terminal Ge_0.81_Sn_0.19_ NW devices after annealing at 523 K for 15, 30, and 60 min. Three different cases of specific device behavior including device failure (α) with breaking at the electrode-NW contact as shown in the inset, resistivity decrease followed by an increase (β) and a steady decrease in resistivity (γ) can be observed. The data presented are from individual devices and not averaged. (b) The XRD of NW material annealed for 60 min at 523 K show Ge_0.81_Sn_0.19_ NW degradation and segregation of β-Sn (ICDD 04-0673). (c) An EDX line scan shows a highly degraded Ge_1–*x*_Sn_*x*_ NW and the corresponding mapping in the inset, which could be associated with α-behavior in (a). A partial segregation is shown in (d) that can be assigned to a β-behavior in (a). The *I*–*V* diagrams in (e) illustrate the β- and γ-behavior of devices in another representation as shown in (a).

In general, electronic devices can be exposed to increased temperatures either by *in situ* joule heating or temperature fluctuations of the whole device. Therefore, we studied 18 Ge_0.81_Sn_0.19_ NWs in two-terminal devices after annealing at 523 K for 15, 30 and 60 min to evaluate their behavior when exposed to temperatures at which segregation processes can be expected (Fig. 4a). Nine of the 18 devices were destroyed after the first heating cycle at 523 K, while only one of these eventually failing devices provides an electronic signal at 15 min annealing with much higher resistivity before the device fails as well at 30 min (α-behavior). The remaining eight devices exclusively show lower resistivity values at room temperature as an indication that the contact resistance was reduced (resistivity in Fig. 4 includes the contact resistance). Four of the surviving devices followed this trajectory and the resistivity decreases further for the whole annealing duration of 60 min (γ-behavior). Another four of the intact devices showed an increased resistivity after annealing for more than 15 min (β-behavior), which can be attributed to the onset of Sn segregation events, while the devices are still intact. An increased resistivity is expected because of the inhomogeneity of the material and lower mobility of Ge_1–*x*_Sn_*x*_ with lower Sn content.^59^ XRD patterns in Fig. 4b of Ge_0.81_Sn_0.19_ NWs treated for 60 min at 523 K show partial material conversion and segregation processes (enlarged in Fig. S1†). In addition to the observed shoulder of the initial Ge_0.81_Sn_0.19_ reflections, small β-Sn reflections can be observed. However, this bulk analysis cannot explain the different behavior of devices observed in the electrical characterization.

To illustrate differences in NWs during this heating process, STEM EDX analysis was performed prior and after the heat treatment at 523 K for 60 min. EDX line scans as well as EDX maps before and after the annealing of the same NWs are illustrated in the ESI† showing completely unaltered NWs, segregation onsets and highly modified NWs (Fig. 4c+d and Fig. S2–4†). The EDX line scan in Fig. 4c illustrates a Ge_1–*x*_Sn_*x*_ NW with pronounced degradation and Sn segregation that could be associated with the device failure (α-behavior), since segregation events are observed in close proximity of the extremes. A partial degradation with the segregation events farther away from the contact material (Fig. 4d) can be responsible for the altered electronic properties resulting in increasing resistivity values (β-behavior). Unaltered Ge_0.81_Sn_0.19_ NWs after heat treatment are associated with γ-behavior and shown in the ESI (Fig. S2†). EDX maps as well as line scans of as-grown and annealed NWs for all three different cases discussed are illustrated in Fig. S2–S4.†

As mentioned before, the here described Ge_0.81_Sn_0.19_ NWs represent a highly metastable material and thus elevated temperatures lead to Sn segregation. The threshold temperature depends on the initial composition including potential doping^61^ and the here described composition should start to degrade at temperatures of ∼523 K.^44^ Formation of metallic Sn particles by segregation from Ge_0.81_Sn_0.19_ NWs is a stochastic process and therefore a difference in device behavior can be expected for the devices. The difference between the completely failing devices and the ones showing only higher resistivity can be related to diffusion length of the segregated Sn in the Ge_0.81_Sn_0.19_ crystal and interactions of the metallic Sn and the Au contacts. Therefore the location of the Sn segregation process is also important. For the device failure upon annealing two scenarios, which are most likely both intertwined, have to be considered for the gap formation at the nanostructure-to-contact region (Fig. 4a). The out-diffusion of Sn upon segregation is accompanied with a Ge_0.81_Sn_0.19_ material conversion to a material with lower Sn content and smaller lattice parameters. This will result in a natural shrinkage of the material and therefore the devices can fail due to mechanical stress indicated by the gap close to the contact in the inset of Fig. 4a. In addition, reaction of the segregated Sn with the Au contact and associated Sn diffusion in the Au contact material can lead to device failure. The probability of this reaction is higher for Sn segregation events in proximity to the Au bond pads leading to failure, while segregation in the middle of a wire does only lead to strain and no structurally weakened NWs. A Au/Sn reaction can cause pronounced formation of pores in diffusion experiments on the Sn side^62^ and the diffusion lengths can be in the tens of nanometer as calculated in the ESI.† 63

## Conclusions

We present the first investigation on the electronic properties of Ge_1–*x*_Sn_*x*_ materials with high Sn content. Ge_0.81_Sn_0.19_ NWs were prepared by microwave synthesis and investigated in two- and four-point configuration demonstrating high conductivity while the contact resistance dominates the *I-V*-characteristics in two-terminal devices. All devices investigated revealed ohmic behavior. The resistivity evolution by cooling to 10 K shows semiconductor characteristics with small increase of resistivity. The thermal annealing at moderate temperatures of 523 K results in three sets of device behavior with influences of the material degradation by Sn segregation on the electronic properties and structural device stability.

## Conflicts of interest

There are no conflicts to declare.

## Supplementary Material

Supplementary information
