# Supplementary material for: Electrical characterization and examination of temperature-induced degradation of metastable Ge0.81Sn0.19 nanowires
Source: Nanoscale. 2018 Oct 12;10(41):19443–9. doi: 10.1039/c8nr05296d (PMC6202951; doi:10.1039/c8nr05296d)
Supplement: Supplementary file 1 [file NR-010-C8NR05296D-s001.pdf]

Electronic Supplementary Material (ESI) for Nanoscale.
This journal is © The Royal Society of Chemistry 2018

# Electronic Supplementary Information

# Electrical Characterization and Examination of Temperature-Induced degradation of Metastable $Ge_{0.81}Sn_{0.19}$ Nanowires

*Masiar Sistani,*[‖,‡] *Michael S. Seifner,*[¥,‡] *Maximilian G. Bartmann,*[‖] *Jürgen Smoliner,*[‖] *Alois Lugstein,*[‖] *Sven Barth*[*,¥]

[¥]TU Wien, Institute of Materials Chemistry, Getreidemarkt 9, 1060 Vienna, Austria.

[‖] TU Wien, Institute of Solid State Electronics, Floragasse 7, 1040 Vienna, Austria.

Description of potential influences on device failure:

There are two scenarios that have to be considered for the device failure upon annealing, which are most likely both responsible for the obtained behavior:

a) The formation of strain due to the ´out-diffusion´ of Sn upon segregation, which is accompanied with a $Ge_{0.81}Sn_{0.19}$ material conversion to a material with lower Sn content. This will result in a natural shrinkage of the material and therefore the devices fail due to mechanical stress.
b) As soon as a Sn segregated segment is in contact with the Au pad, Sn diffusion in the Au ´bulk´ material can lead to failure of the device. It is known from diffusion experiments that Au/Sn reaction can lead to pronounced formation of pores in on the Sn side [1]

In addition, the diffusion length can be estimated /calculated according $x^2 \cong 4Dt$ using diffusion data from literature and the correlation of diffusivity ($D = D_0 \exp(-\frac{Q}{RT})$). For the tracer diffusion coefficient of Sn in Au, $D_0 = 4.1\times10^{-6}$ m²/s and Q = 143 kJ/mol are reported at T = 974–1273 K. [2]

This leads to a diffusion length of $x \cong 8.8$ nm with R = 8.314 J/(mol · K) and $t$=900 s (15 min), while $x \cong 17.6$ nm for 60 min.

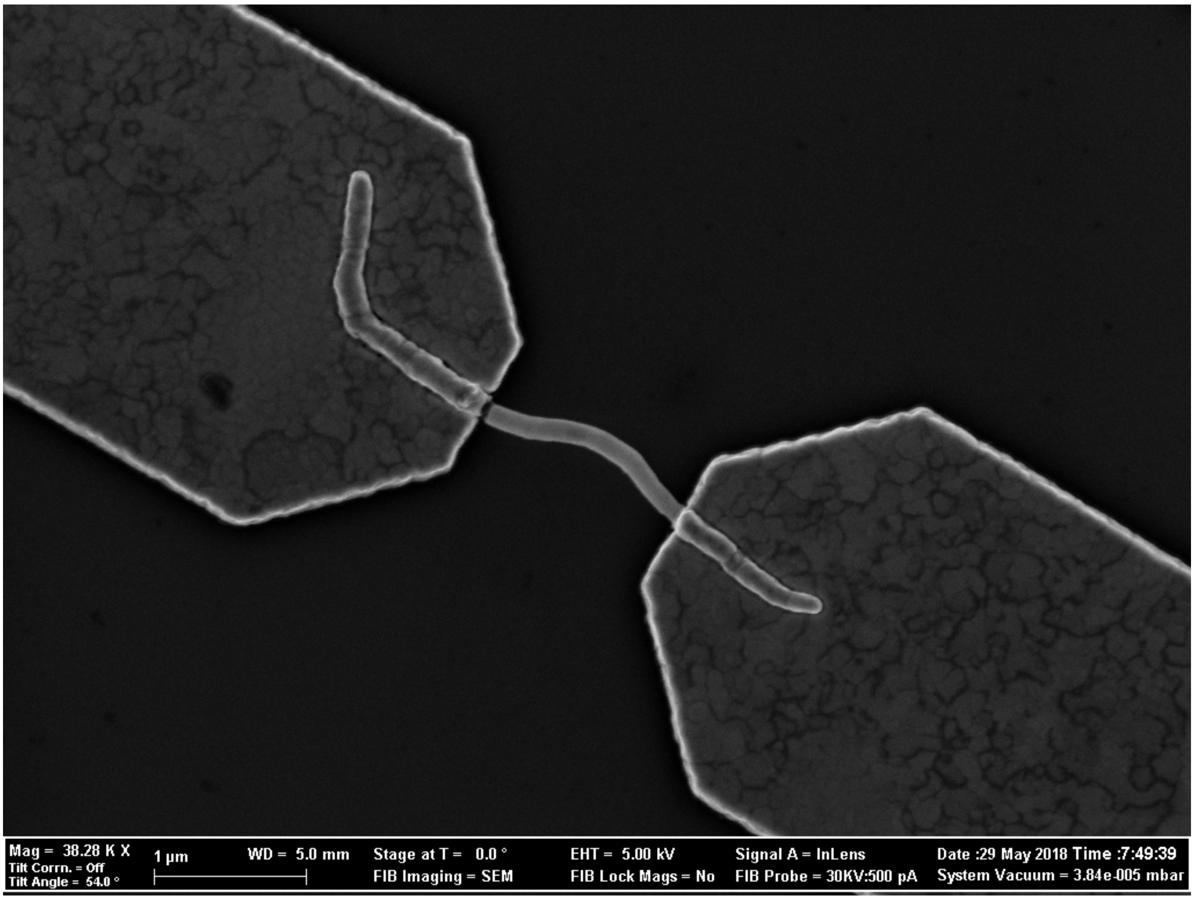

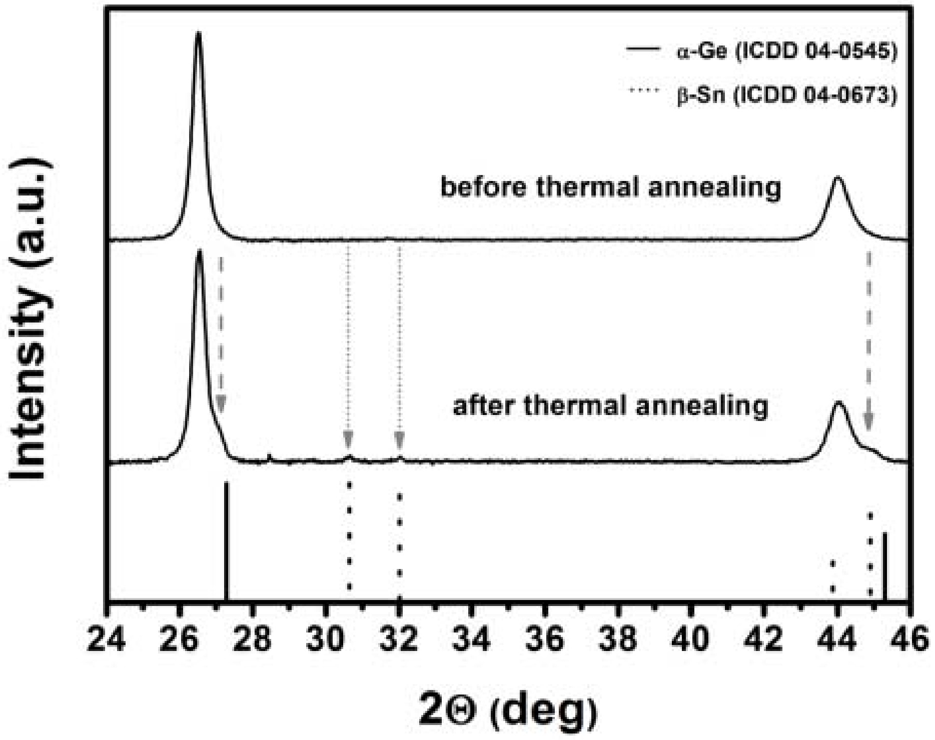


Figure S1: The XRD pattern of as-grown $Ge_{0.81}Sn_{0.19}$ NWs after removal of metallic Sn by HCl treatment shows a single phase, which allows to calculate the chemical composition being 18.8% Sn according to Vegard's law. Annealing the material for 1h at 250 °C leads to a partial segregation process of Sn, which is indicated by the arrows(· · · >) and a secondary $Ge_{1-x}Sn_x$ phase (x<0.19; fitted to be x= 0.07) marked by (- - >).

As-grown:

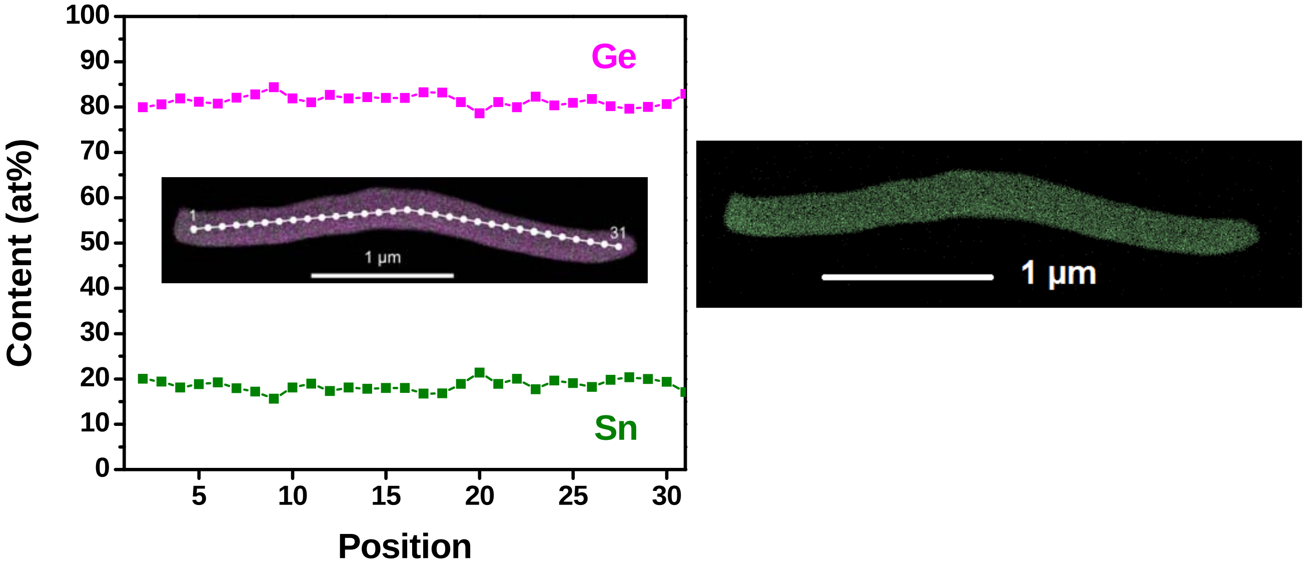


Annealed for 60 min at 250 °C:

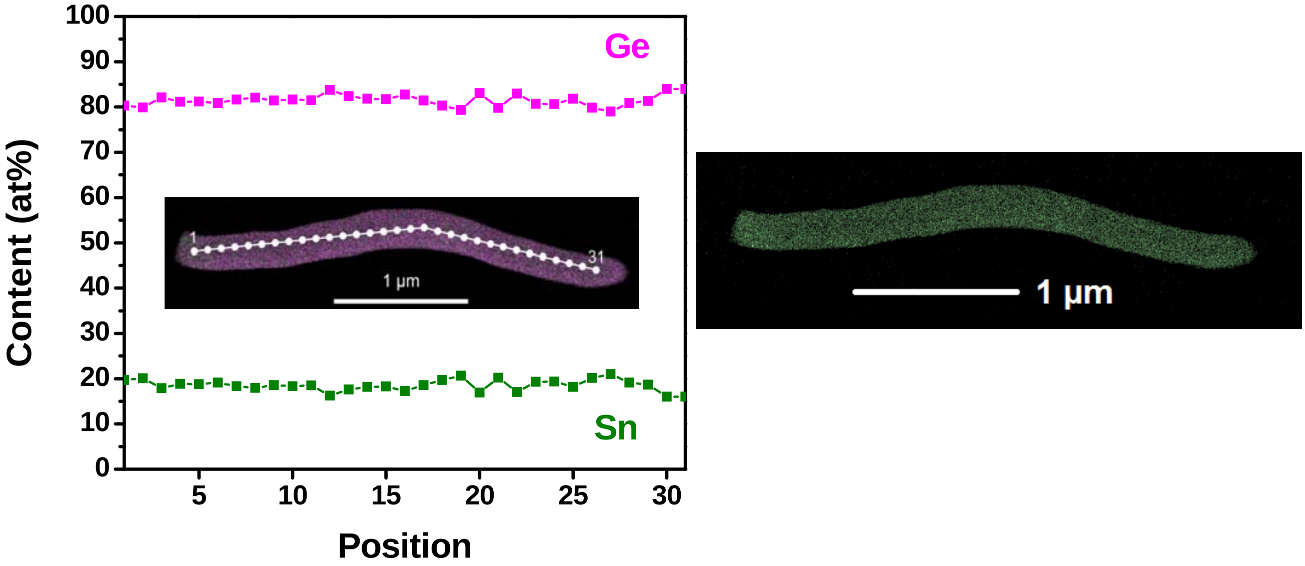


Figure S2: EDX line scans and Sn maps for the same $Ge_{0.81}Sn_{0.19}$ NW as-grown and annealed at 523 K for 60 min. There is no evidence for material degradation.

As-grown:

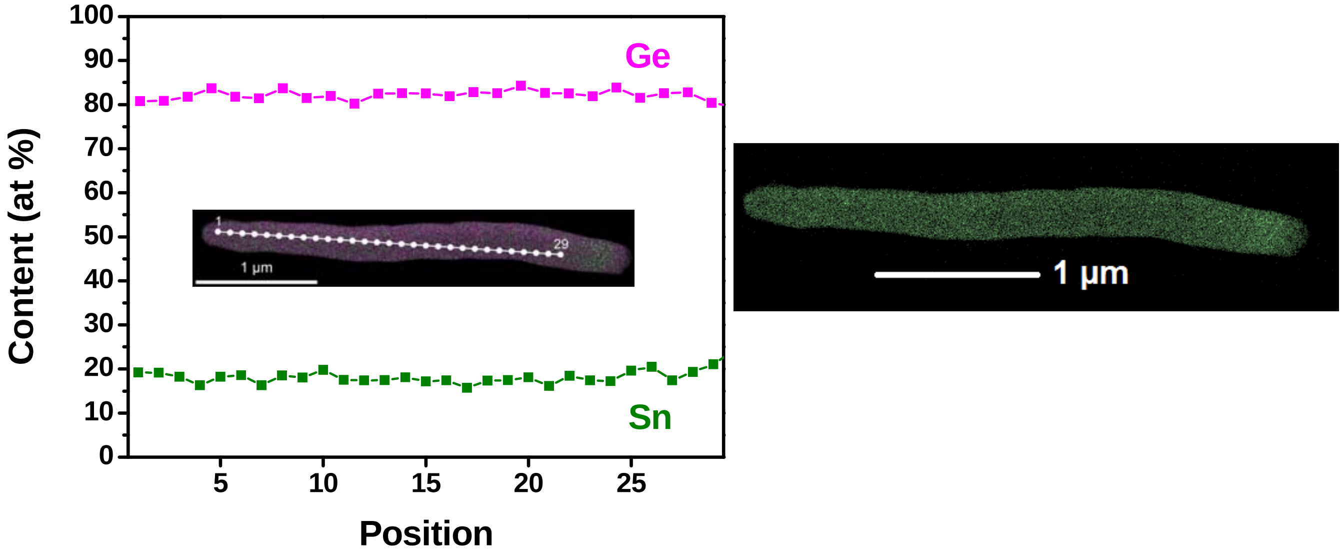


Annealed for 60 min at 250 °C:

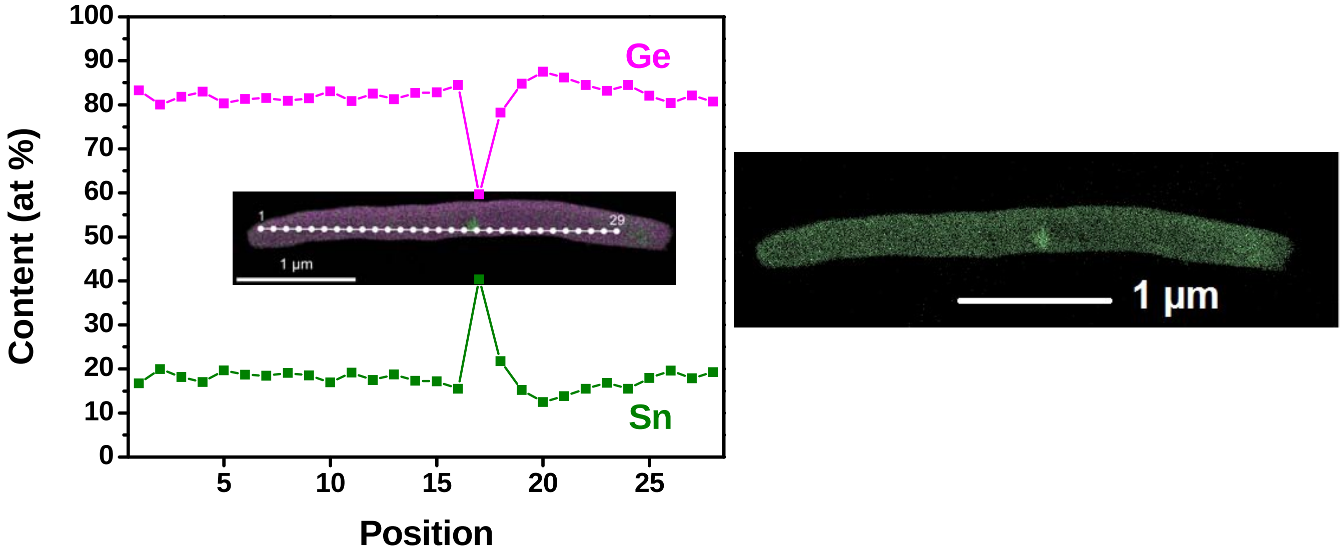


Figure S 3: EDX line scans and Sn maps for the same $Ge_{0.81}Sn_{0.19}$ NW as-grown and annealed at 523 K for 60 min. A small fraction of the NW material shows degradation/segregation evidenced by an enriched Sn area.

As-grown:

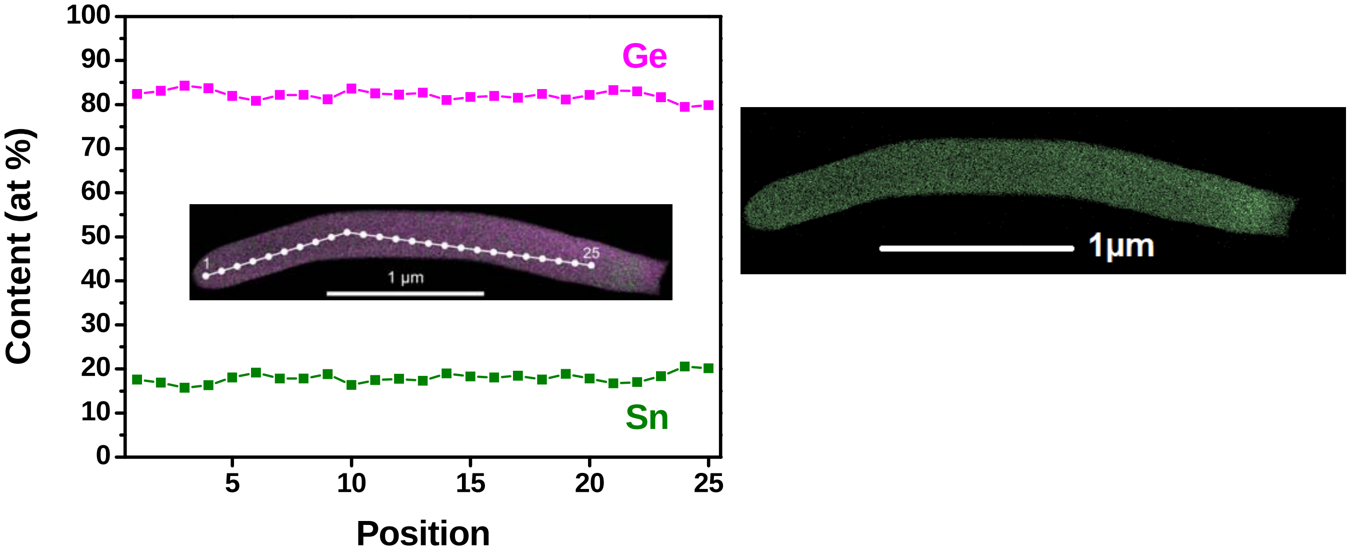


Annealed for 60 min at 250 °C:

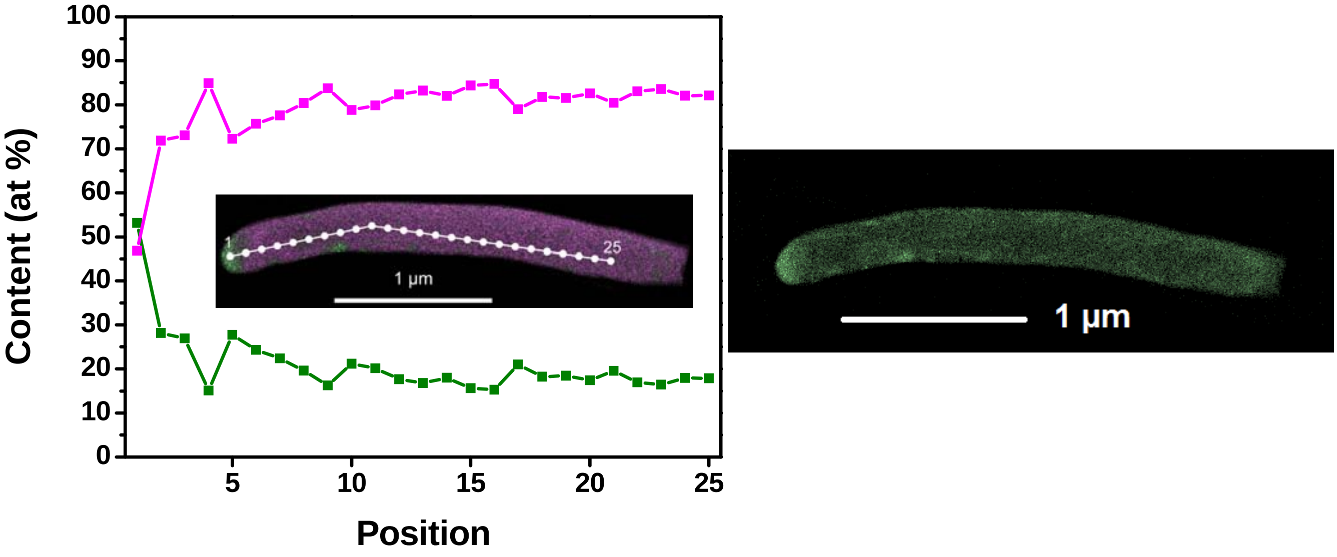


Figure S 4: EDX line scans and Sn maps for the same $Ge_{0.81}Sn_{0.19}$ NW as-grown and annealed at 523 K for 60 min. A significant degree of degradation and Sn segregation after annealing is evident in the line scan as well in the tin map, which could lead to device failure.

# References:

(1) Ghosh, C. *Intermetallics* **2010**, *18*, 2178.
(2) Yamada, T.; Miura, K.; Kajihara, M.; Kurokawa, N.; Sakamoto, K. *Materials Science and Engineering: A* **2005**, *390*, 118.
